# Osteoinductive and Biocompatibility Assessment of a 3D-Printed Polymeric–Hydroxyapatite Composite Interference Screw

**DOI:** 10.3390/polym18101239

**Published:** 2026-05-19

**Authors:** Rana Smaida, Louis-Paul Maugard, Hervé Gegout, Manuel Arruebo, Florence Fioretti, Nadia Benkirane-Jessel, Henri Favreau

**Affiliations:** 1 Institut National de la Santé et de la Recherche Médicale (INSERM) UMR1260, Nanomédicine Régénérative, 1 Rue Eugène Boeckel, 67000 Strasbourg, France; lp.maugard@chu-tours.fr (L.-P.M.); herve.gegout@unistra.fr (H.G.); henri.favreau@chru-strasbourg.fr (H.F.); 2 Faculté de Pharmacie, Faculté de Chirurgie Dentaire, Faculté de Médecine, Université de Strasbourg, 4 Rue Blaise Pascal, 67000 Strasbourg, France; 3 Instituto de Nanociencia y Materiales de Aragón (INMA), CSIC-Universidad de Zaragoza, 50009 Zaragoza, Spain; arruebom@unizar.es; 4 Department of Chemical Engineering, University of Zaragoza, Campus Río Ebro-Edificio I + D, C/Poeta Mariano Esquillor S/N, 50018 Zaragoza, Spain

**Keywords:** 3D printing, polymer-hydroxyapatite composite, interference screw, osteoinduction, biocompatibility, tendon graft fixation, preclinical model

## Abstract

Anterior cruciate ligament reconstruction relies on interference screw fixation, yet insufficient graft osseointegration remains a critical clinical challenge. This study aimed to develop and characterize a 3D-printed polymeric–hydroxyapatite composite interference screw with an osteoinductive surface to enhance localized osteogenic responses. Screws were designed, modeled, and fabricated using fused deposition modeling 3D printing with a polycaprolactone-poly(lactic-co-glycolic acid)-hydroxyapatite composite. Physico-chemical characterization was performed using scanning electron microscopy. Biocompatibility was assessed through mesenchymal stem cell metabolic activity assays and morphological analysis. Osteogenic gene expression was quantified by RT-qPCR following culture in osteogenic differentiation medium. In vivo osseointegration was evaluated histologically at five and nine weeks following implantation in the proximal tibial epiphysis of a rat model. 3D printing successfully produced screws with consistent geometry and surface characteristics. The composite material supported robust mesenchymal stem cell proliferation without cytotoxicity or morphological abnormalities. Histological examination revealed progressive bone formation with no adverse tissue reactions, including the absence of cyst formation, osteolysis, or excessive fibrosis. RT-qPCR revealed upregulation of osteogenic markers in those enhanced screws. These results indicate that the 3D-printed polymeric–hydroxyapatite composite screws are biocompatible and capable of stimulating localized osteogenic activity, supporting their potential as a biological foundation for future evaluation in anterior cruciate ligament reconstruction applications.

## 1. Introduction

Anterior cruciate ligament (ACL) rupture represents a major and urgent clinical challenge in orthopedic trauma and sports medicine [1]. It is one of the most frequent ligamentous injuries requiring surgical intervention and has become an increasing public health concern due to rising participation in physical activity and sports across all age groups [2]. ACL injuries not only affect athletic performance but also have long-term implications for knee stability, function, and quality of life [3,4,5].

### 1.1. Epidemiology, Clinical Significance and Economic Burden

ACL injury incidence in Europe ranges from 29–51 per 100,000 people per year, with rising trends particularly among females and younger age groups [6], and approximately 200,000 individuals are affected annually in the United States [7]. These injuries impose a substantial economic and social burden that extends well beyond direct surgical costs [8,9,10]. Population-level economic modeling in athletic cohorts suggests that the mean cost per ACL injury event, including treatment and potential future sequelae, can exceed $30,000 [11] with higher costs for non-operative management when accounting for work absence, loss of earnings, and long-term disability [12]. Direct and indirect costs of ACL injuries collectively exceed $7 billion per year [13]. Current research also highlights that return to pre-injury sport remains variable, with reinjury rates around 20–30% [14]. These epidemiological and economic data underscore the need for improved reconstruction strategies.

### 1.2. Surgical Reconstruction: Current Strategies and Limitations

The ACL’s primary biomechanical role is to limit anterior tibial translation and control rotational movements of the knee, functions that are essential for dynamic activities [15]. The ACL’s hierarchical collagen organization provides high tensile strength but limited mid-substance vascularity, contributing to its poor intrinsic healing capacity after rupture [16]. A successful healing response following injury or reconstruction requires both tendon graft integration within bone tunnels and intra-articular remodeling (ligamentization), processes that are inherently slow and often incomplete [17]. For these reasons, surgical reconstruction remains the standard of care for symptomatic instability in active individuals. Reconstruction involves placement of autografts through femoral and tibial bone tunnels, with fixation devices such as interference screws used to achieve immediate structural stability [18]. Despite widespread use, ACL reconstruction failure rates range from 3.2% to 11.1%, with tunnel malposition, traumatic reinjury, patient-related factors, and biological limitations in graft healing identified as major contributors [19]. Current grafts fail to fully replicate the biological and biomechanical characteristics of the native ACL, with autografts and allografts both demonstrating limitations in vascularization, remodeling, and mechanical fidelity [20,21]. These data highlight a critical unmet need for strategies that enhance biological graft integration, particularly at the graft–bone interface, to improve reconstruction durability, accelerate recovery, and optimize long-term clinical outcomes [22]. Most critically, the graft–bone interface represents the weakest link of the reconstruction construct, with insufficient osseointegration as the primary determinant of early graft failure and the principal target for therapeutic intervention [17,23,24]. These gaps have driven the development of strategies integrating cellular, molecular, and material-based approaches to enhance graft–bone healing.

### 1.3. Emerging Therapies

In response to the persistent limitations of conventional ACL reconstruction, increasing research efforts have focused on biological augmentation strategies aimed at enhancing graft healing and regeneration beyond mechanical fixation alone [25]. Rather than relying on mechanical fixation only, these strategies seek to incorporate osteoconductive and osteoinductive cues into fixation devices to enhance cellular recruitment, bone deposition, and graft incorporation [26]. By accelerating biological incorporation, such approaches aim to reduce early failure rates and support return to sport, highlighting the need for next-generation fixation devices combining mechanical support with active tissue regeneration.

#### 1.3.1. Biological Augmentation Strategies

Among these approaches, platelet-rich plasma (PRP), mesenchymal stem cells (MSCs), and growth factor-based therapies have been extensively investigated. PRP has demonstrated the capacity to promote early graft maturation and vascularization in preclinical models and early clinical studies [27,28,29,30].

MSC-based therapies derived from bone marrow, tendon, vascular, or umbilical cord sources have similarly shown potential to enhance ligamentization and tendon-bone healing in experimental settings [30,31,32,33]. Growth factors play a central regulatory role in tissue repair by stimulating cell proliferation, matrix synthesis, and neovascularization, thereby orchestrating the early stages of ligament healing [13,34]. Among growth factor therapies, bone morphogenetic protein-2 (BMP-2) has been investigated for its osteoinductive properties making it an attractive possible candidate for enhancing graft–bone osseointegration in ACL reconstruction [32,35,36,37].

Preclinical studies have demonstrated that localized BMP-2 delivery via biomaterial carriers may support bone formation and tendon-to-bone healing [37,38]. Combined strategies, including BMP-2 overexpression in MSCs with basic fibroblast growth factor (bFGF), have enhanced osteogenic differentiation and tendon-bone healing through Smad pathway activation more effectively than monotherapy [39]. However, despite these biological advantages, this application remains limited by significant translational challenges [40,41].

#### 1.3.2. Biomaterial-Based Strategies and Additive Manufacturing

To address both biological and mechanical limitations of current reconstruction strategies, biomaterial-based approaches have emerged as a rational platform for enhancing graft–bone integration [42]. Biodegradable polymers such as polycaprolactone (PCL) and poly(lactic-co-glycolic acid) (PLGA) are widely used in orthopedic applications due to their biocompatibility, tunable mechanical properties, and controllable degradation profiles [43,44]. PCL provides high tensile strength and slow degradation (several months in vivo), supporting load-bearing applications, [45] while PLGA degrades more rapidly due to the higher hydrophilicity of composing glycolic acid compared to that of lactic acid [46] and has an established clinical safety profile, both with U.S. Food and Drug Administration (FDA) approval for multiple clinical applications.

The incorporation of hydroxyapatite (HA), a naturally occurring calcium phosphate mineral, further enhances osteoconductivity by providing nucleation sites for bone mineral deposition and improves mechanical stability through composite reinforcement [47].

In vitro studies have demonstrated the biocompatibility and osteogenic potential of PCL, PLGA and HA composite scaffolds in various tissue engineering applications [48,49,50,51,52,53,54]. PCL/HA composite scaffolds support attachment, proliferation, and osteogenic differentiation of multiple MSC populations, as evidenced by elevated ALP activity and osteogenic gene expression [55]. The addition of PLGA to PCL/HA composites improves mechanical properties and biocompatibility, while its faster degradation creates in situ porosity favorable for cellular infiltration and matrix remodeling [48]. Approximately 40% w/w HA has been identified as optimal in PCL-based matrices, maximizing compressive modulus while preserving porosity; beyond this threshold, uneven HA aggregation weakens interfacial bonding and reduces mechanical strength [56,57]. In addition, biomimetic scaffold systems, including aligned gelatin microribbon hydrogels incorporating HA gradients, have shown capacity to guide zonal MSC differentiation and partially reproduce the native bone–tendon interface architecture, though mechanical insufficiency and lack of translational evidence remain limitations [58]. However, these constructs have not yet been translated beyond experimental settings. Electrospun PCL/HA/collagen composite scaffolds have similarly shown suitable mechanical properties, controlled degradation, and cytocompatibility [59]. Recent advances in additive manufacturing enable precise control over implant geometry, porosity, composition, and degradation kinetics, allowing fabrication of mechanically robust, patient-specific devices incorporating bioactive molecules [60,61]. This integration of mechanical design and biological functionality represents a critical step toward clinically viable regenerative fixation systems, though scaffolds alone remain insufficient to fully recapitulate the hierarchical organization, biological activity, and functional integration of the native ACL. Polyelectrolyte multilayer coatings on orthopedic implant surfaces have similarly demonstrated the capacity to promote osseointegration and angiogenesis in vivo [62].

### 1.4. Translational Barriers and Limitations of Current Strategies

Despite strong preclinical evidence, clinical translation of biological augmentation strategies has proven challenging, requiring biological efficacy, safety, reproducibility, regulatory feasibility, and cost-effectiveness, with most strategies remaining in early-phase evaluation with limited long-term data. PRP is the most extensively studied biological adjunct, yet multiple systematic reviews and meta-analyses of RCTs have demonstrated no clinically meaningful improvements in patient-reported outcomes or knee stability beyond one year, with substantial heterogeneity in preparation and delivery methods limiting reproducibility [63,64]. MSC-based therapies have progressed slowly due to regulatory, manufacturing, and logistical challenges. A phase Ib/IIa trial in 17 patients demonstrated safety of allogeneic mesenchymal precursor cells following ACL reconstruction with improved Knee Injury and Osteoarthritis Outcome Score (KOOS) scores at 24 months, though the primary focus was osteoarthritis prevention rather than graft healing [65]. A separate exploratory study of umbilical cord blood-derived MSCs in 27 patients reported no adverse events but no significant differences in tunnel enlargement, laxity, or functional scores at two years [66]. Overall, no adequately powered RCT has yet demonstrated the clinical superiority of MSC-based augmentation over standard reconstruction.

Growth factor therapies face substantial translational barriers due to regulatory scrutiny and safety concerns. Among these, BMP-2 has shown osteoinductive potential in preclinical models of tendon-bone healing [32,67,68] but despite FDA approval for spinal fusion (anterior lumbar interbody fusion) and open tibial fractures, it has not yet been evaluated in prospective clinical trials specifically targeting ACL reconstruction, representing a critical gap between its well-documented preclinical efficacy and clinical translation [69]. Nevertheless, clinical translation of BMP-2 has revealed significant safety concerns. In spinal fusion, high-dose rhBMP-2 on absorbable collagen sponges has been associated with serious adverse events at high doses, including heterotopic ossification, osteolysis, and inflammatory reactions [70], underscoring the importance of delivery strategies that minimize systemic exposure. Advanced delivery strategies have focused on biomaterial-based surface coatings enabling cell-contact-dependent bioavailability, in which molecules are presented at the material surface and made available through direct cell contact rather than passive diffusion or burst release, allowing localized biological signaling while limiting systemic exposure [71,72,73,74,75,76,77,78,79,80,81]. The coating strategy employed in the present study is based on this principle, representing a modular surface addition adaptable to other bioactive molecules rather than a systemic or high-dose delivery system. Biomaterial-based interference screws with osteoconductive coatings have reached clinical use through biocompatibility and mechanical equivalence pathways, with most lacking prospective trials demonstrating superior osseointegration. Limited high-quality clinical evidence reflects regulatory complexity, outcome measure ceiling effects, and economic barriers, highlighting the need for therapies demonstrating meaningful improvements in patient-centered outcomes to justify adoption.

### 1.5. Existing Commercial Solutions and Limitations

A range of commercial products has been developed for ACL repair and ligament regeneration, spanning biological scaffolds, synthetic ligaments, and augmentation systems, each with distinct advantages and limitations. Biological scaffolds such as the Bridge-Enhanced ACL Repair (BEAR^®^) Implant, BioBrace^®^, and OrthoPure^®^ XT aim to support native tissue healing by providing a collagen-rich matrix that promotes cellular infiltration and regeneration [82,83,84,85]. Synthetic ligaments and reinforcement devices offer immediate mechanical strength to support load-bearing while acting as structural substitutes or adjuncts to the native ligament. Additionally, augmentation and fixation systems like InternalBrace and FiberTag^®^ TightRope^®^ provide reinforcement during or after reconstruction to enhance stability and graft fixation [86,87]. Despite these innovations, current commercial solutions face challenges including mismatched degradation rates, insufficient mechanical properties, and limited biological modulation capacity, underscoring the need for next-generation biomaterials combining mechanical support with progressive biological integration, enabled by additive manufacturing.

### 1.6. Study Objective and Novelty

Building on these insights and addressing the biological limitations of current fixation devices, we developed a 3D-printed interference screw composed of PCL, PLGA, and hydroxyapatite as the primary focus of this study, with the composite formulation itself designed to provide the core mechanical and osteoconductive properties required for graft fixation. A modular bioactive surface based on a cell-contact-dependent BMP-2 nanoreservoir coating, developed and patented by our group, was applied to assess whether localized osteoinductive signaling could further support the osteogenic potential; in this system, BMP-2 bioavailability is restricted to direct cell-surface contact rather than passive release. The modular nature of the coating further allows incorporation of other bioactive molecules depending on the target clinical indication. The novelty of this approach resides not in any single component in isolation but in their specific integration: the ternary PCL + PLGA + HA (30/30/40) composition was empirically validated through a systematic three-arm biological comparison demonstrating its superiority over binary alternatives, and the combination of this formulation with a cell-contact-dependent BMP-2 nanoreservoir coating within a single FDM-fabricated interference screw has not been previously reported. To determine the optimal biomaterial composition, three polymer formulations were systematically compared: Group 1 (PCL + HA, 60/40), Group 2 (PCL + PLGA, 50/50), and Group 3 (PCL + PLGA + HA, 30/30/40). The optimal formulation was subsequently produced in two versions: Implant A, incorporating a surface coating, and Implant B, a non-coated control, which were used for all subsequent biological and in vivo evaluations. Biocompatibility was assessed in vitro using mesenchymal stem cell cultures, providing foundational evidence for cytocompatibility. The implant was characterized by scanning electron microscopy. Osteogenic potential was evaluated via RT-qPCR quantification of differentiation markers (alkaline phosphatase, osteocalcin, osteopontin), and in vivo osseointegration was examined using a rat tibial implantation model with histological analysis at five and nine weeks after implantation, capturing both early and intermediate phases of bone integration. These results provide a proof-of-concept foundation for a biocompatible and osteoconductive composite interference screw with a modular bioactive surface coating, adaptable to other bioactive molecules, and representing a promising platform for next-generation regeneration fixation devices.

## 2. Materials and Methods

### 2.1. Materials

Polycaprolactone (PCL) (pellets, Mw. 45,000 Da) was obtained from Sigma Aldrich (St. Louis, MO, USA). Poly(lactic-co-glycolic acid) (PLGA) (50:50, Resomer® RG504, Mw. = 7000–17,000 Da) was obtained from Evonik Industries, GmbH (Darmstadt, Germany). Human bone marrow-derived mesenchymal stem cells (hBM-MSCs) and Mesenchymal Stem Cell Growth Medium 2 were obtained from PromoCell (Heidelberg, Germany). α-Minimum essential medium (α-MEM) and Paraformaldehyde was obtained from Gibco^®^, ThermoFischer Scientific, Illkirch, France. Hydroxyapatite nanoparticles (particle size < 200 nm ≥97%, synthetic), 2-(N-Morpholino)ethanesulfonic acid (MES) buffer, Hexamethyldisilazane (HMDS), Ascorbic acid, Phosphate-buffered saline (PBS), β-glycerophosphate, and dexamethasone were obtained from Sigma-Aldrich (St. Louis, MO, USA). Chitosan (Protasan UPCL 113, 500 μg/mL) was obtained from NovaMatrix (Sandvika, Norway). Recombinant human BMP-2 was obtained from Euromedex (Souffelweyersheim, France). AlamarBlue reagent was obtained from Invitrogen (Waltham, MA, USA). TRIzol reagent was obtained from Invitrogen. High-Capacity cDNA Reverse Transcription Kit was obtained from Applied Biosystems (Foster City, CA, USA). SYBR Green Master Mix was obtained from Applied Biosystems. Hematoxylin and eosin were obtained from Merck (Darmstadt, Germany) and Molekula (Dorset, UK), respectively. Gomori Trichrome reagents, including ferric hematoxylin (Groat’s iron hematoxylin) and the acid dye mixture (Chromotrope 2R, Light Green SF, and phosphotungstic acid), were obtained from Acros Organics (Geel, Belgium), Merck (Darmstadt, Germany), Alfa Aesar (Haverhill, MA, USA), and Carlo Erba Reagents (Val-de-Reuil, France). Alizarin Red S was obtained from Carl Roth GmbH (Karlsruhe, Germany). Eukitt synthetic resin (Carlo Erba Reagents, Val-de-Reuil, France) was used as the mounting medium for all histological preparations. Ethylenediaminetetraacetic acid (EDTA) was obtained from Euromedex (Souffelweyersheim, France). Vicryl resorbable sutures were obtained from Ethicon (Raritan, NJ, USA). Wistar rats were obtained from Charles River Laboratories (Saint-Germain-sur-l’Arbresle, France).

### 2.2. Methods

#### 2.2.1. Development of Interference Screws by 3D Printing

Screw macrostructure characterization and structural parameter determination were based on a review of biomechanical studies. Optimal screw characteristics for clinical applications were defined as follows: length 7 mm in length, diameter 5 mm, conical longitudinal design, thread depth 0.8 mm, trapezoidal thread profile with proximal half-angle 30°, and thread pitch 1.6 mm. A reduced-scale model was subsequently designed for rat implantation and histological analysis. Due to limitations in printing resolution and anatomical constraints of the rat tibial epiphysis, only the screw head was fabricated for implantation studies. Three-dimensional printing was performed using fused deposition modeling with a 0.25 mm extrusion nozzle diameter. Computer modeling was executed using Marlin Firmware version 1.1.6, and G-code generation was performed using Cura® version 3.0 software (Ultimaker B.V., Utrecht, The Netherlands). Polymer filaments for screw fabrication were prepared by extrusion from PCL pellets and PLGA powder. An original Prusa i3 MK3S printer was used to fabricate the screws. Three different compositions were produced to evaluate material properties and biological performance: Group 1 (PCL + HA, 60/40), Group 2 (PCL + PLGA, 50/50), and Group 3 (PCL + PLGA + HA, 30/30/40). Hydroxyapatite nanoparticles (particle size < 200 nm) were incorporated to enhance osteoconductivity and promote cellular differentiation toward the osteoblastic lineage. The screws contained a microporous core with approximately 400 μm pore size to promote cell infiltration and proliferation.

#### 2.2.2. Physicochemical Characterization of Three-Dimensional Printed Screws

Scanning electron microscopy examination of screw microstructure and surface morphology was performed using a Hitachi S800 scanning electron microscope (Hitachi, Tokyo, Japan) operated at 15 kV accelerating voltage. Samples were mounted on aluminum stubs and sputter-coated with gold under vacuum to ensure electrical conductivity. Energy-dispersive X-ray spectroscopy was performed to confirm hydroxyapatite distribution throughout the composite material and to verify elemental composition.

#### 2.2.3. Sterilization and Sample Preparation

Sterilization of interference screws was performed by sequential immersion in two successive baths of 70% ethanol for 15 min each, followed by ultraviolet irradiation for 30 min. This procedure was repeated twice to ensure complete sterilization. Following sterilization, screws were aseptically sectioned using sterile scalpel blades to obtain uniform samples for in vitro and in vivo experiments. All subsequent handling was performed under sterile conditions in a laminar flow biosafety cabinet.

#### 2.2.4. Mesenchymal Stem Cell Culture

Human bone marrow-derived mesenchymal stem cells were obtained from PromoCell (Heidelberg, Germany) and cultured in Mesenchymal Stem Cell Growth Medium MSC2 according to the manufacturer’s instructions. Bone marrow-derived MSCs represent the gold-standard reference population for in vitro osteogenic differentiation assays, given their well-characterized multilineage differentiation potential [88] and their superior osteogenic commitment compared to MSCs derived from other tissue sources under standard differentiation conditions [89].

Cells were maintained in tissue culture flasks at 37 °C in a humidified atmosphere containing 5% CO_2_. At confluence, MSCs were trypsinized for seeding on the implants. Cells were rinsed with PBS, then incubated with 2.5 mL of trypsin for 5 min at 37 °C. 5 mL culture medium was added subsequently and centrifuged at 220× *g* for 4 min and 30 s. Following centrifugation, cells were resuspended in fresh culture medium and counted using a hemocytometer. Cells were seeded at equal density on samples previously placed in 48-well plates, with glass coverslips serving as two-dimensional control substrates. After three days of culture in proliferation medium, cells on implants were transferred to osteogenic differentiation medium consisting of α-minimum essential medium supplemented with 60 μM ascorbic acid, 10 mM β-glycerophosphate, and 0.1 μM dexamethasone. Osteogenic differentiation culture was maintained for 21 days at 37 °C in a humidified atmosphere with 5% CO_2_.

#### 2.2.5. Scanning Electron Microscopy of Cell-Seeded Biomaterials

Scanning electron microscopy observation was performed to assess cell morphology and distribution on implant surfaces. Following 21 days of culture in osteogenic differentiation medium, cell-seeded biomaterials were fixed with 4% paraformaldehyde solution for 10 min at 4 °C. Samples were then dehydrated using successive ethanol baths of 20 min each at increasing concentrations (50%, 70%, 90%, 100% ethanol). Final dehydration was completed with a three-minute immersion in 100% hexamethyldisilazane solution. Samples were then mounted on aluminum stubs and sputter-coated with gold. Observations were performed using a Hitachi S800 scanning electron microscope at 15 kV accelerating voltage. Multiple fields of view were examined for each sample at magnifications ranging from 40× to 3500×.

#### 2.2.6. Cellular Metabolic Activity Measurement by Alamar Blue

Cellular metabolic activity was assessed using the AlamarBlue assay (Invitrogen), which is based on the reduction of resazurin (blue, non-fluorescent) to resorufin (pink, fluorescent) by metabolically active cells. Measurements were performed 3, 7, 14, and 21 days after the initiation of osteogenic differentiation culture. At each time point, samples were transferred to fresh 48-well plates and incubated with 400 μL of AlamarBlue solution (10% *v*/*v* in culture medium) for 4 h at 37 °C in the dark. Following incubation, 100 μL of supernatant from each well was transferred to a 96-well plate, and fluorescence intensity was measured at 590 nm emission wavelength (530 nm excitation) using a microplate reader (Tecan, Männedorf, Switzerland). Background fluorescence was determined from control wells containing only culture medium and AlamarBlue reagent without cells. Metabolic activity was calculated as fluorescence intensity normalized to background controls. Experiments were performed in triplicate.

#### 2.2.7. Bone Morphogenetic Protein-2 Surface Coating

BMP-2 was incorporated onto the implant surface using a polyelectrolyte multilayer deposition technique. This approach is designed to present the bioactive molecule at the material surface in a cell-contact-dependent manner, enabling localized biological availability upon direct cell-surface interaction rather than passive diffusion or burst release. This nanoreservoir technology has been patented by our group (WO2012/113812; EP11305182) and extensively validated across multiple tissue engineering applications with BMP-2 and other bioactive molecules [71,72,79]. Sterilized implants were placed in flat-bottom 48-well plates prior to coating.

Implant B served as control. Surface coating was performed via polyelectrolyte multilayer assembly. Implants were first immersed three times for 5 min each in 2-(N-morpholino)ethanesulfonic acid (MES) buffer (pH 5.5), followed by a 15 min immersion in a chitosan solution (0.5 mg/mL in MES buffer, pH 5.5). After three additional 5 min rinses in MES buffer, the implants were immersed for 15 min in BMP-2 solution (200 ng/mL in phosphate-buffered saline, PBS). This sequence constituted one coating cycle, which was repeated six times to build a multilayer structure. Following the final cycle, implants were rinsed three times with sterile PBS and air-dried under sterile conditions.

Following surface coating, confirmation of coating deposition was performed by scanning electron microscopy as described above, comparing Implant A and Implant B surfaces at multiple magnifications (40×, 100×, 1000×). Morphological differences in surface topography were documented to verify successful coating application.

#### 2.2.8. RNA Extraction and RT-qPCR

Following 14 and 21 days of culture in osteogenic differentiation medium, total RNA was extracted from cells cultured on biomaterials using TRIzol reagent (Invitrogen) according to the manufacturer’s protocol. Briefly, samples were lysed in TRIzol reagent, and RNA was isolated by chloroform extraction and isopropanol precipitation. RNA pellets were washed with 75% ethanol, air-dried, and resuspended in nuclease-free water. RNA concentration and purity were assessed by spectrophotometry using a NanoDrop instrument (Thermo Scientific). Complementary DNA synthesis was performed using the High-Capacity cDNA Reverse Transcription Kit (Applied Biosystems, Foster City, CA, USA) with 1 μg of total RNA. Reverse transcription was carried out according to the manufacturer’s instructions.

Quantitative polymerase chain reaction was performed using SYBR Green Master Mix (Applied Biosystems) on a StepOnePlus Real-Time PCR System (Applied Biosystems). The following genes were analyzed: alkaline phosphatase (ALP), osteocalcin (OCN), Bone Gamma-Carboxyglutamate Protein (BGLAP), bone sialoprotein 2 (BSPII), and runt-related transcription factor 2 (RUNX2). Glyceraldehyde 3-phosphate dehydrogenase (GAPDH) was used as a housekeeping gene for normalization. Each sample was analyzed in triplicate. Relative gene expression was calculated using the 2^−ΔΔCt^ method.

#### 2.2.9. Animal Model and Surgical Procedure

All animal procedures were performed in accordance with European Union Directive 2010/63/EU for the protection of animals used for scientific purposes and were approved by the institutional ethics committee APAFIS#7740-2016112116448550 (20 September 2016) and APAFIS#26926-2020080410295545 (20 December 2020).

In accordance with the 3R principles for the ethical use of animals in research, the sample size was minimized while retaining sufficient animals to assess biocompatibility and obtain preliminary histological evidence of osseointegration. This experiment was designed exclusively as a proof-of-concept biocompatibility and osseointegration assessment and does not simulate ACL reconstruction, tendon-bone tunnel healing, graft fixation, or joint loading conditions. A total of 8 animals were used. Animals were randomly assigned to receive either Implant A (surface-coated, n = 4) or Implant B (uncoated control, n = 4). Each group was further subdivided for sacrifice at five weeks (n = 2) or nine weeks (n = 2) post-implantation, yielding n = 2 per group per timepoint.

Surgical procedures were performed under aseptic conditions. Animals were anesthetized with isoflurane (maintenance 2–3% in oxygen). A medial parapatellar incision was made, and subcutaneous tissues were dissected to expose the joint capsule. The patella was carefully luxated laterally to expose the proximal tibial epiphysis. A standardized bone defect (2 mm diameter) was created in the proximal tibial epiphysis using a dental drill under constant sterile saline irrigation. The biomaterial sample was press-fitted into the defect. The patella was repositioned, and the joint capsule was closed with resorbable sutures (Vicryl, Ethicon). Animals received postoperative analgesia (buprenorphine 0.05 mg/kg) for 48 h. Animals were monitored daily for signs of infection or distress.

#### 2.2.10. Sample Collection and Histological Processing

Animals were euthanized at five weeks (n = 4, 2 per group) and nine weeks (n = 4, 2 per group) post-implantation by carbon dioxide inhalation followed by cervical dislocation. The proximal tibial epiphysis containing the implant was carefully dissected, cleaned of soft tissues, and immediately fixed in 4% paraformaldehyde solution for 48 h at 4 °C. Following fixation, samples were demineralized in 10% ethylenediaminetetraacetic acid solution (EDTA, pH 7.4) for four weeks at 4 °C with solution changes performed twice weekly. Demineralized samples were dehydrated through graded ethanol series, cleared in xylene, and embedded in paraffin wax. Serial sections (5 μm thickness) were cut using a rotary microtome (Leica RM2255, Leica Biosystems, Wetzlar, Germany) and mounted on glass slides. Sections were stained with hematoxylin and eosin for general morphological evaluation. Briefly, sections were deparaffinized in xylene, rehydrated through graded ethanol series, stained with hematoxylin for five minutes, differentiated in acid alcohol, blued in ammonia water, stained with eosin for two minutes, dehydrated, cleared, and mounted with permanent mounting medium. For mineralization assessment, additional serial sections were stained with Alizarin Red S to visualize calcium deposition and mineralized bone matrix. Briefly, deparaffinized and rehydrated sections were rinsed in distilled water, stained with 2% Alizarin Red S solution (pH 4.1–4.3, adjusted with ammonium hydroxide) for 2–5 min at room temperature, rinsed in distilled water, dehydrated rapidly through ascending acetone concentrations, cleared in acetone-xylene (1:1) followed by xylene, and mounted. Mineralized bone and calcium deposits appear bright red under this staining protocol. For collagen organization and tissue composition assessment, Gomori Trichrome staining was performed on additional serial sections. Sections were deparaffinized, rehydrated through graded ethanol series, fixed in Bouin’s solution overnight at room temperature, washed in running tap water until yellow color disappeared, stained in Weigert’s iron hematoxylin working solution for 10 min, rinsed in distilled water, differentiated in 1% phosphomolybdic-phosphotungstic acid solution for 10–15 min, stained directly (without rinsing) in aniline blue solution for 5–10 min, rinsed briefly in distilled water, differentiated in 1% acetic acid solution for 2–5 min, dehydrated through graded ethanol series, cleared in xylene, and mounted. In this staining protocol, collagen fibers appear blue/green, nuclei appear black, cytoplasm appears red, and bone matrix demonstrates variable blue-green coloration depending on mineralization status. Histological examination was performed using an optical microscope (Olympus BX53, Olympus Corporation, Tokyo, Japan) at magnifications of 4×, 10×, and 20×. Digital images were captured using a CCD camera (Olympus DP73, Olympus Corporation, Tokyo, Japan). Histological evaluation focused on bone–implant interface characteristics, new bone formation, inflammatory response, fibrous tissue formation, vascularization, and material integration. Specific attention was directed toward the identification of woven versus lamellar bone morphology, presence and distribution of osteocytes within lacunae, bone marrow organization, inflammatory cell infiltration, and vascular structures.

#### 2.2.11. Statistical Analysis

Statistical analysis was performed using GraphPad Prism version 9.0 (GraphPad Software, San Diego, CA, USA). Data are presented as mean ± standard deviation (SD) for RT-qPCR data and mean ± standard error of the mean (SEM) for metabolic activity assays. Normality of data distribution was assessed using the Shapiro–Wilk test, and homogeneity of variances was assessed using Levene’s test; parametric tests were applied when both assumptions were met (Shapiro *p* > 0.05; Levene *p* > 0.05). For comparisons between two groups, Student’s *t*-test (for normally distributed data) or Mann–Whitney U test (for non-normally distributed data) was employed. For multiple group comparisons over time, two-way analysis of variance (ANOVA) including the interaction term, followed by Tukey post hoc multiple comparisons with Bonferroni correction, was used. For RT-qPCR data, statistical differences were evaluated gene-by-gene using a two-way ANOVA with Time_Point (Day 14 vs. Day 21) and Condition (Implant A vs. Implant B) as factors, including the interaction term (Time_Point × Condition), followed by Tukey/emmeans post hoc multiple comparisons with Bonferroni correction. Statistical significance was defined as * *p* < 0.05, ** *p* < 0.01, *** *p* < 0.001. All experiments were performed with appropriate biological replicates and technical replicates as specified in the individual method sections.

## 3. Results

### 3.1. Production and Structural Characterization of 3D-Printed Interference Screws

To develop a fixation device capable of providing both mechanical support and osteoinductive stimulation at the graft–bone interface, three polymer composite formulations were designed and fabricated by fused deposition modeling (FDM): Group 1 (PCL + HA, 60/40), Group 2 (PCL + PLGA, 50/50), and Group 3 (PCL + PLGA + HA, 30/30/40). The primary objective of this first set of experiments was to confirm that the 3D printing process could reproducibly yield screws with the geometry, surface characteristics, and internal architecture required for subsequent in vitro and in vivo evaluation.

**Figure 1:**
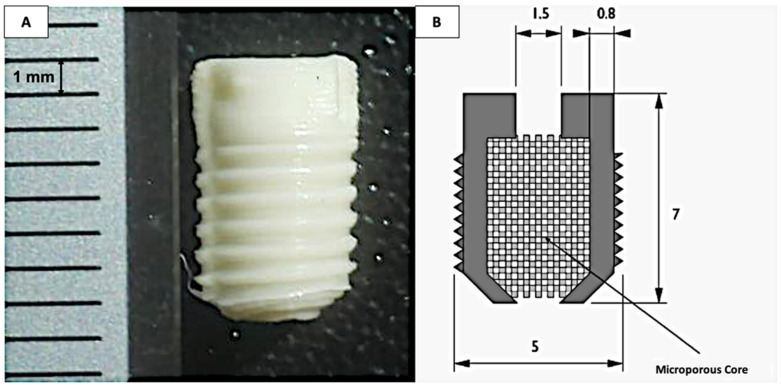
3D-printed polymeric–hydroxyapatite composite interference screw head with integrated microporous core. (**A**) Representative photograph of the screw head shown alongside a millimeter scale. (**B**) Schematic cross-sectional view illustrating the screw geometry and dimensions (in millimeters). The central core consists of a microporous lattice with an average pore size of approximately 400 µm.

All three formulations were successfully fabricated with reproducible geometry consistent with computer-aided design specifications (Figure 1A,B). Macroscopic examination confirmed well-defined thread patterns and uniform surface morphology across all groups. The printed screw heads measured approximately 7 mm in length with a 5 mm maximum diameter. All compositions incorporated a central microporous core with an average pore size of approximately 400 µm (Figure 1B), designed to facilitate cell infiltration and vascularization following implantation. Energy-dispersive X-ray spectroscopy verified homogeneous distribution of the commercial hydroxyapatite particles throughout the polymer matrix in Groups 1 and 3, with calcium and phosphorus peaks confirming the presence of hydroxyapatite phase (Ca/P molar ratio ≈ 1.7) within the composite material.

Taken together, these results established that the FDM process successfully produced structurally consistent composite screws across all formulations, providing a reproducible material platform for comparative biological evaluation.

### 3.2. Cell Attachment and Morphology on Biomaterial Surfaces: SEM Assessment

**Figure 2:**
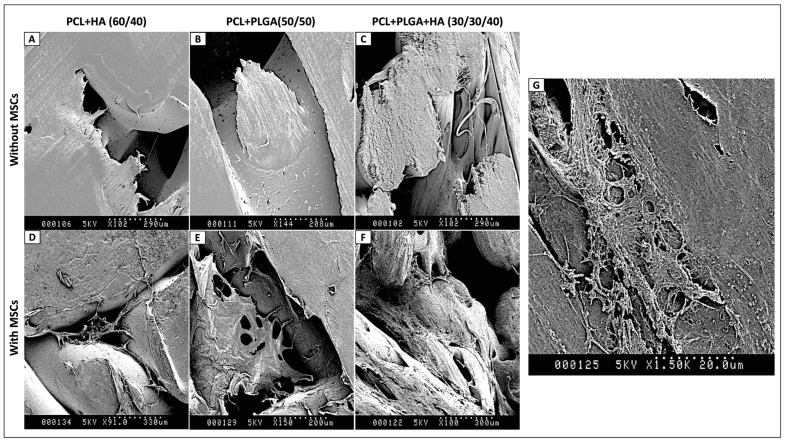
Scanning electron microscopy characterization of mesenchymal stem cell attachment and proliferation on three biomaterial compositions. Top panels (**A**–**C**): Biomaterial surfaces prior to cell seeding, showing the characteristic surface morphology of Group 1 (PCL + HA, 60/40) (**A**), Group 2 (PCL + PLGA, 50/50) (**B**), and Group 3 (PCL + PLGA + HA, 30/30/40) (**C**) respectively. Bottom panels (**D**–**F**): Mesenchymal stem cells after 21 days of culture in osteogenic differentiation medium, demonstrating robust cell attachment, spreading, and surface colonization on Groups 1 (**D**), 2 (**E**), and 3 (**F**), respectively. Panel (**G**): Higher magnification view (×3500) illustrating characteristic elongated morphology of healthy, proliferative mesenchymal stem cells with extensive filopodia and lamellipodia formation extending across the Group 3 biomaterial surface, indicating strong cell–substrate interactions and active synthetic activity.

Having established structural reproducibility, the next step was to assess whether each composite formulation could support mesenchymal stem cell (MSC) adhesion and surface colonization. The objective of this experiment was therefore to evaluate cell attachment, spreading, and three-dimensional infiltration on all three biomaterial compositions following 21 days of culture in osteogenic differentiation medium, using scanning electron microscopy (SEM) as a morphological readout of cell–substrate interaction. SEM examination of MSC-seeded surfaces revealed robust cell attachment and colonization across all three compositions (Figure 2). Prior to cell seeding, surfaces of Groups 1, 2, and 3 displayed the characteristic layer-by-layer printing texture, with smooth polymer surfaces and occasional micropores inherent to the FDM process (Figure 2A–C). After 21 days of osteogenic culture, cells on all three formulations exhibited the characteristic elongated morphology of healthy, proliferative MSCs in active synthetic phases (Figure 2D–F and panel G enlarged view). Extensive filopodial and lamellipodial extensions were observed across the material surfaces, indicative of strong cell–substrate interactions. Cell distribution was relatively homogeneous, with confluent cell layers in many regions, and evidence of three-dimensional scaffold infiltration into pore spaces was clearly noted, suggesting active colonization of the interior architecture rather than surface-only attachment. No morphological abnormalities were detected in any group. High-magnification imaging at ×3500 of Group 3 (PCL + PLGA + HA) confirmed well-developed cytoskeletal spreading with multiple filopodia (Figure 2G), consistent with strong integrin-mediated adhesion to the composite surface. These morphological data suggested the cytocompatibility of all three formulations and provided a qualitative basis for subsequent quantitative metabolic activity measurements.

### 3.3. Comparative Metabolic Activity of Mesenchymal Stem Cells: Selection of the Optimal Composite Formulation

**Figure 3:**
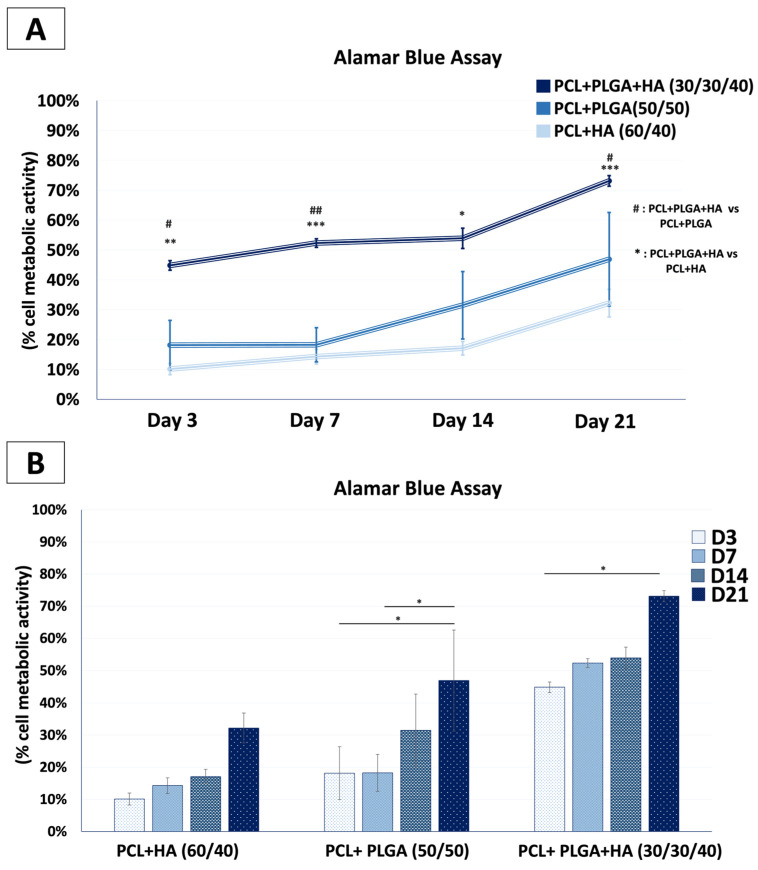
Alamar Blue assay evaluating mesenchymal stem cell metabolic activity on three 3D-printed biomaterial compositions over time. (**A**) Line graph showing percentage of cell metabolic activity measured at days 3, 7, 14, and 21 on PCL + HA (60/40), PCL + PLGA (50/50), and PCL + PLGA + HA (30/30/40) substrates. Days 3, 7, 14, and 21 refer to the duration of osteogenic differentiation culture, defined as Day 0 of osteogenic induction initiated after 3 days of proliferation medium pre-culture. (**B**) Corresponding bar graph representation of metabolic activity for each composition at individual time points (D3, D7, D14, D21). Data are presented as mean ± SEM (n = 3 biological replicates per group per timepoint). Statistical annotations indicate comparisons between material groups as follows: * comparison between PCL + PLGA + HA and PCL + HA; # comparison between PCL + PLGA + HA and PCL + PLGA in Figure 3A. Normality of residuals was assessed using the Shapiro–Wilk test, and homogeneity of variances was assessed using Levene’s test; assumptions were met for all groups (Shapiro *p* > 0.05; Levene *p* > 0.05). Statistical differences were evaluated by two-way ANOVA, followed by Tukey multiple comparisons with Bonferroni correction.#: PCL + PLGA + HA vs. PCL + PLGA (# *p* < 0.05, ## *p* < 0.01, (n = 3 per group)) *: PCL + PLGA + HA vs. PCL + HA (*p* < 0.05 (*), *p* < 0.01 (**), *p* < 0.001 (***)).

While SEM provided qualitative evidence of cell attachment across all formulations, a quantitative assessment of cellular viability and proliferative activity was required to identify the most supportive composition for subsequent biological studies. The Alamar Blue assay was therefore employed to measure MSC metabolic activity on all three composite formulations at days 3, 7, 14, and 21 of osteogenic differentiation culture, enabling longitudinal comparison and selection of the optimal biomaterial composition. Two complementary representations of the data are presented in Figure 3: a line graph (Figure 3A) illustrating the longitudinal metabolic trajectory of each formulation over the 21-day culture period, and a bar graph (Figure 3B) enabling direct side-by-side comparison of all three materials at each individual timepoint. Together, these representations capture both the kinetics of the cellular response and the magnitude of between-group differences at discrete observation points.

As early as day 3, cells on PCL + PLGA + HA (30/30/40) and PCL + PLGA (50/50) demonstrated significantly higher metabolic activity (44.2 ± 2.1% and 43.8 ± 1.8%, respectively) compared to PCL + HA (60/40) (18.7 ± 3.4%; *p* < 0.003 and *p* < 0.024, respectively) (Figure 3). This early advantage was sustained throughout the culture period: at day 7, PCL + PLGA + HA maintained superior activity (52.1 ± 1.2%) compared to PCL + HA (15.8 ± 5.3%; *p* < 0.0013). The most pronounced differences were observed at day 21, at which point PCL + PLGA + HA reached 73.5 ± 1.8%, significantly exceeding both PCL + PLGA (53.6 ± 9.2%; *p* < 0.0006) and PCL + HA (31.8 ± 8.3%; *p* < 0.026). Notably, PCL + PLGA + HA demonstrated the steepest progressive increase, achieving a 1.66-fold rise from day 3 to day 21, suggesting a synergistic effect among the three components: PCL contributes to the mechanical stability and slow degradation, PLGA enhances surface hydrophilicity and porosity to improve cellular access, and HA provides osteoconductive nucleation sites that support cell anchorage and metabolic activity. These results established PCL + PLGA + HA (30/30/40) as the superior composite formulation and justified its selection for all subsequent studies.

### 3.4. Confirmation of Surface Coating Deposition by Scanning Electron Microscopy

Having selected PCL + PLGA + HA (30/30/40) as the optimal formulation, the next objective was to apply a modular bioactive surface coating and to confirm the structural success of the deposition procedure prior to biological validation. Scanning electron microscopy was therefore performed to characterize the surface topography of PCL + PLGA + HA scaffolds before and after deposition of the coating.

**Figure 4:**
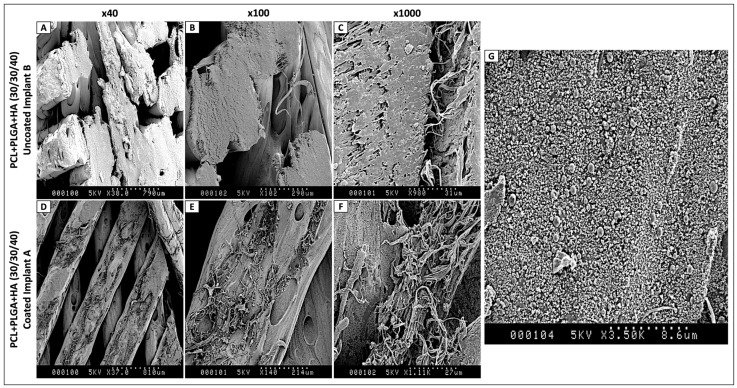
Scanning electron microscopy characterization of surface coating deposition on PCL + PLGA + HA (30/30/40). Panels (**A**–**C**): *Implant B* control implant surfaces at increasing magnifications (40×, 100×, 1000×) showing the characteristic texture of the 3D-printed polymer-hydroxyapatite composite with relatively smooth surfaces and occasional micropores resulting from the fused deposition modeling printing process. Panels (**D**–**F**): Implant A surfaces at corresponding magnifications (40×, 100×, 1000×) demonstrating distinct morphological changes compared to Implant B controls. Small vesicle-like structures and coating aggregates are visible on the material surface. Panel (**G**): At 3500× magnification, the coating appears as a discontinuous particulate layer with characteristic nodular morphology distributed across the substrate surface, indicating a successful coating.

Implant B displayed the characteristic morphology of FDM-fabricated constructs, with relatively smooth polymer-hydroxyapatite surfaces, well-defined printing layer patterns, and occasional intrinsic micropores at higher magnifications (Figure 4A–C). Following six cycles of multilayer deposition, substantial and consistent morphological modifications were observed across all magnifications (Figure 4D–F). At ×40, the coated surfaces exhibited altered overall texture with visible surface organic deposits compared to Implant B controls. At ×100, small vesicle-like structures and coating aggregates were clearly distributed across the material surface. At ×1000, distinct particulate formations and increased surface roughness attributable to the bioactive coating were apparent. Ultra-high magnification at ×3500 (Figure 4G) further revealed the coating architecture as a discontinuous particulate layer with characteristic nodular morphology distributed across the substrate. These observations confirm successful deposition of the multilayer coating onto the PCL + PLGA + HA surface, with preserved underlying porosity potentially favorable for cell attachment and sustained bioactive molecule release. This structural validation provided the essential prerequisite for meaningful interpretation of the downstream RT-qPCR and in vivo data.

Despite the fact that three successive washing steps with sterile PBS were carried out, SEM observations confirmed that the layer-by-layer process followed produced a strong adhesion between the morphogenetic protein and the chitosan-based coating.

### 3.5. Coating Accelerates Early Osteogenic Gene Expression: RT-qPCR Analysis

**Figure 5:**
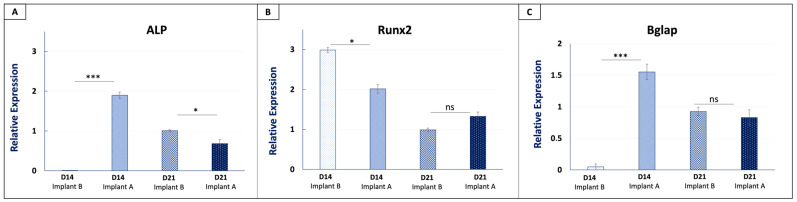
Relative gene expression of osteogenic markers in MSCs cultured on Implant A and Implant B 3D-printed PCL + PLGA + HA (30/30/40) composite scaffolds at days 14 and 21 of osteogenic differentiation. Days 14 and 21 refer to the duration of osteogenic differentiation culture, initiated after 3 days of proliferation medium pre-culture. Implant A: surface-coated PCL + PLGA + HA (30/30/40); Implant B: uncoated PCL + PLGA + HA (30/30/40) control. (**A**) Alkaline phosphatase (ALP), (**B**) Runt-related transcription factor 2 (RUNX2), and (**C**) Bone gamma-carboxyglutamate protein (BGLAP). Gene expression was normalized to GAPDH using the 2^−ΔΔCt^ method. Data are presented as mean ± SD (n = 4 biological replicates, each analyzed with three technical replicates). Normality of residuals was assessed using the Shapiro–Wilk test, and homogeneity of variances was assessed using Levene’s test; assumptions were met for all genes (Shapiro *p* > 0.05; Levene *p* > 0.05). Statistical differences were evaluated gene-by-gene using a two-way ANOVA (Time_Point: Day 14 vs. Day 21; Condition: Implant A vs. Implant B) including the interaction term (Time_Point × Condition), followed by Tukey/emmeans post hoc multiple comparisons with Bonferroni correction. Significance is indicated as follows: *p* < 0.05 (*) and *p* < 0.001 (***).

Morphological confirmation of the coating was a necessary but not sufficient demonstration of its biological activity. The critical question was whether the deposited coat retained its osteoinductive capacity and could activate the transcriptional programs associated with osteoblastic differentiation. Quantitative RT-PCR was therefore performed to evaluate the temporal expression of key osteogenic markers, ALP, RUNX2, and BGLAP in MSCs cultured on Implant A versus Implant B PCL + PLGA + HA scaffolds at days 14 and 21 of osteogenic differentiation (Figure 5).

At day 14, Implant A induced a significant upregulation of ALP compared to Implant B controls (*p* < 5.72 × 10^−7^), consistent with early osteoblastic activation (Figure 5A). ALP is a well-established early marker of osteogenic differentiation, typically peaking during the matrix maturation phase before declining as mineralization progresses [90,91]. By day 21, ALP expression was significantly higher in Implant B controls relative to Implant A (*p* < 0.013), a pattern consistent with the expected temporal decline following peak induction in Implant A, reflecting progression toward an advanced differentiation stage in the coated scaffolds.

RUNX2, the master transcription factor of osteoblast differentiation, was significantly elevated in Implant B controls compared to Implant A at day 14 (*p* < 0.028) while no significant difference was observed between groups at day 21 (Figure 5B). RUNX2 is known to be rapidly and transiently induced following osteogenic stimulation [90], and its apparent elevation in Implant B controls at day 14 could reflect a delayed transcriptional induction relative to Implant A, in which RUNX2 activation may have already peaked and begun to decline before the day 14 sampling point. This temporal offset is coherent with osteogenic coat accelerating the early transcriptional program, with the osteogenic cascade already progressing toward later stages by day 14 in Implant A.

BGLAP expression at day 14 was significantly higher in Implant A compared to Implant B controls (*p* < 4.05 × 10^−6^), indicating enhanced early commitment to matrix mineralization (Figure 5C). BGLAP encodes osteocalcin, a non-collagenous bone matrix protein produced by mature osteoblasts that is directly involved in hydroxyapatite binding. By day 21, BGLAP levels in both groups reached comparable levels without statistically significant difference, consistent with progressive osteogenic maturation in both conditions [91].

Overall, these results suggest that the surface coating retained biological activity and was associated with earlier osteogenic marker expression at day 14, most notably ALP and BGLAP, consistent with an acceleration of the osteoblastic transcriptional program at this early timepoint. By day 21, all markers reached similar expression levels across both conditions, reflecting the natural convergence of both conditions toward a mature osteoblastic phenotype at this later timepoint [92]. These in vitro findings prompted us to investigate whether this accelerated osteogenic commitment would translate into enhanced bone formation and mineralization in vivo.

### 3.6. In Vivo Osseointegration and Biocompatibility Study

The in vitro data collectively established the cytocompatibility and osteoconductive potential of the PCL + PLGA + HA composite, and suggested that the surface coating of Implant A was associated with earlier osteogenic marker expression. To determine whether these properties translated to an accelerated osseointegration response in a living system, an in vivo study was conducted using a rat tibial implantation model. The primary objectives were: (i) to confirm the absence of adverse tissue reactions in both coating conditions across the full observation period, thereby establishing the safety profile of the implants; and (ii) to obtain preliminary histological evidence of osseointegration. This experiment does not represent an ACL reconstruction model. Three complementary histological staining modalities hematoxylin and eosin (H&E), Gomori Trichrome, and Alizarin Red S were employed to provide a complete assessment of tissue morphology, collagen organization, and mineralization, respectively.

The medial parapatellar surgical approach provided reliable access to the proximal tibial epiphysis across all animals, with consistent identification of anatomical landmarks and standardized creation of 2 mm diameter bone defects. All implants were successfully placed and specimens retrieved at 5 and 9 weeks post-implantation (n = 2 per group per timepoint) with adequate preservation of the bone–implant interface. Serial sections (5 µm) were processed for staining.

#### 3.6.1. H&E Histological Assessment: Biocompatibility and Bone–Implant Integration

**Figure 6:**
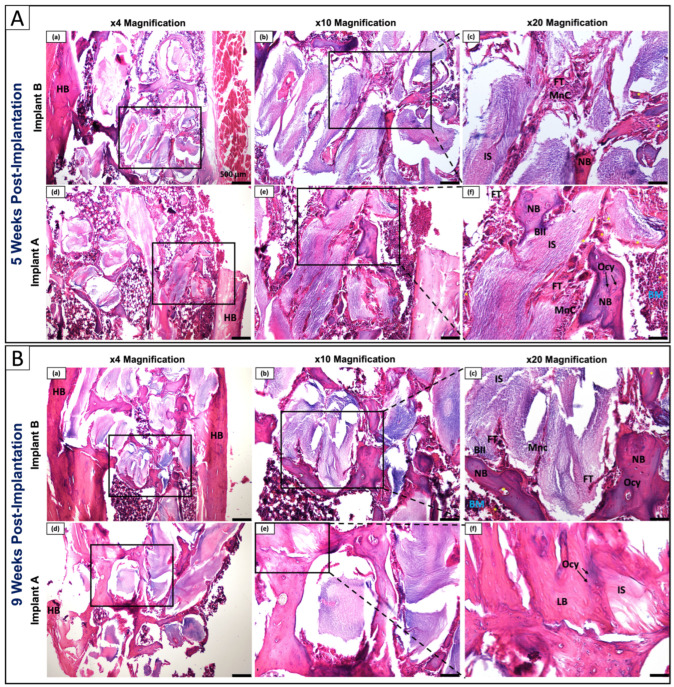
Histological evaluation of bone–implant interface following implantation of Implant A and Implant B 3D-printed interference screw heads in the proximal tibial epiphysis of rats. Representative hematoxylin and eosin (H&E)-stained sections are shown at low (×4), intermediate (×10), and high (×20) magnifications. (**A**) Five weeks post-implantation: panels (**a**–**c**) correspond to Implant B, and panels (**d**–**f**) correspond to Implant A. (**B**) Nine weeks post-implantation: panels (**a**–**c**) correspond to Implant B, and panels (**d**–**f**) correspond to Implant A. Black rectangles indicate regions shown at higher magnification. Abbreviations: IS, interference screw; FT, fibrous tissue; NB, newly formed bone; LB, lamellar bone; BII, bone–implant interface; Ocy, osteocytes within lacunae; BM, bone marrow; MnC, mononuclear inflammatory cells; blood sinusoids are indicated by asterisks (*). Observation timepoints: 5 weeks (**A**) and 9 weeks (**B**) post-implantation. Scale bar: 500 μm.

H&E staining was performed to evaluate tissue response, biocompatibility, and bone–implant interface morphology at 5 and 9 weeks post-implantation in the proximal tibial epiphysis of rats (Figure 6). Across all conditions and timepoints, implants demonstrated biocompatibility. No cyst formation, osteolytic lesions, or pathological fibrosis was observed, no giant cells in response to a foreign material were observed either, and the inflammatory response remained limited to mononuclear cells (MnC) consistent with physiological remodeling, without acute neutrophilic or chronic granulomatous reactions, confirming the safety profile of the implants in both coating conditions, directly corroborating the in vitro Alamar Blue and SEM biocompatibility data.

Beyond biocompatibility, H&E evaluation revealed a progressive osseointegration response that was enhanced by surface coating. At 5 weeks, Implant B demonstrated moderate osseointegration, with fibrous tissue (FT) and MnC interposed at the bone–implant interface (BII), and newly formed bone (NB) displaying immature woven architecture in the peri-implant region, with osteocytes (Ocy) embedded within lacunae and scattered blood sinusoids, identifiable by its irregular, disorganized collagen fiber arrangement and randomly distributed osteocyte lacunae characteristic of primary bone formation (Figure 6A(a–c)), consistent with an early remodeling phase. Implants A at the same timepoint showed a trend toward enhanced NB formation with closer apposition to the implant surface (IS), reduced FT interposition, osteocytes well-embedded within lacunae, organized bone marrow spaces (BM), and homogeneous bone matrix infiltration; scattered blood sinusoids were visible within the newly formed bone, consistent with early vascular support of the osseointegration response (Figure 6A(d–f)), suggesting a possible contribution of the surface coating to early osseointegration. By 9 weeks, Implant B showed progressive bone maturation with increased NB formation, though FT and MnC remained visible at the BII alongside developing BM spaces, and early lamellar bone (LB) architecture was observed, characterized by the emergence of more parallel collagen fiber organization in portions of the newly formed bone, though full lamellar maturation was not yet complete; blood sinusoids and developing bone marrow spaces were present (Figure 6B(a–c)), suggesting continued but incomplete integration. Implant A at 9 weeks showed apparent advanced osseointegration: well-organized LB was observed in direct apposition to the IS, displaying the characteristic parallel lamellar architecture with well-defined osteon-like organization, osteocytes were fully integrated within lacunae, abundant BM spaces were present, vascularised and organized, fibrous tissue at the interface was minimal, and clear evidence of matrix infiltration was noted (Figure 6B(d–f)). The transition from woven to lamellar bone and the establishment of direct bone-to-implant contact reflect progressive tissue remodeling, and provide preliminary histological evidence of advanced bone maturation in Implant A compared to Implant B at this timepoint.

#### 3.6.2. Collagen Remodeling and Tissue Maturation Assessed by Gomori Trichrome

**Figure 7:**
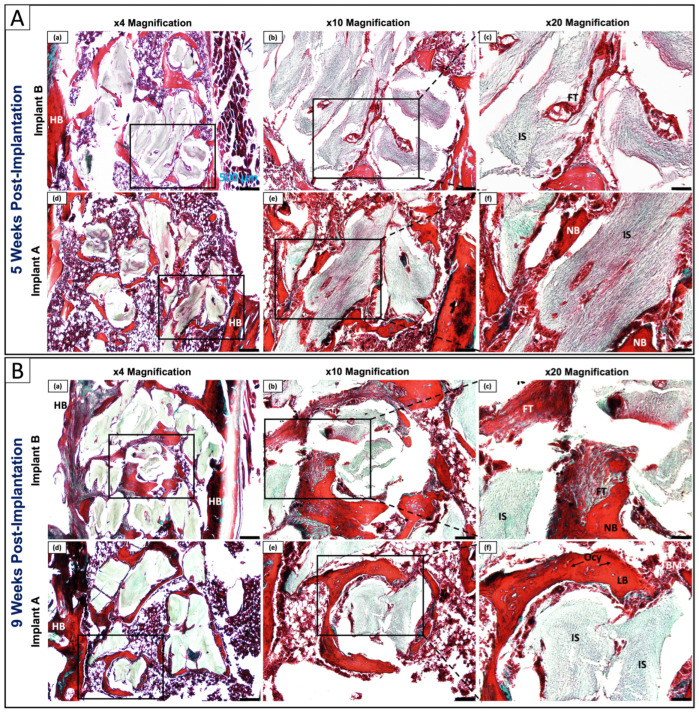
Histological evaluation of bone–implant interface following implantation of Implant A and Implant B 3D-printed interference screw heads in the proximal tibial epiphysis of rats. Representative Gomori Trichrome-stained sections are shown at low (×4), intermediate (×10), and high (×20) magnifications. Representative images demonstrate collagen organization and tissue composition at the bone–implant interface. Collagen fibers appear blue/green, nuclei appear black, and cytoplasm appears red. (**A**) Five weeks post-implantation: panels (**a**–**c**) correspond to Implant B, and panels (**d**–**f**) correspond to Implant A. (**B**) Nine weeks post-implantation: panels (**a**–**c**) correspond to Implant B, and panels (**d**–**f**) correspond to Implant A. Black rectangles indicate regions shown at higher magnification. Abbreviations: IS, interference screw; FT, fibrous tissue; NB, newly formed bone; LB, lamellar bone; BII, bone–implant interface; Ocy, osteocytes within lacunae; BM, bone marrow; MnC, mononuclear inflammatory cells; blood sinusoids are indicated by asterisks (*). Observation timepoints: 5 weeks (**A**) and 9 weeks (**B**) post-implantation. Scale bar: 500 μm.

To characterize the composition and organizational maturity of the peri-implant tissue beyond general morphology, Gomori Trichrome staining was performed to distinguish fibrous collagen (blue-green staining) from mineralized matrix (red staining) at the bone–implant interface (Figure 7). This approach allowed assessment of the rate at which immature fibrous tissue was being replaced by organized, mineralized bone, a key determinant of osseointegration quality.

At 5 weeks, Implant B exhibited heterogeneous tissue composition: host bone (HB) appeared predominantly red, while the peri-implant region displayed mixed blue-green and red staining consistent with incompletely mineralized collagen matrix (Figure 7A(a–c)). Fibrous tissue formed a distinct layer adjacent to the IS, with predominantly disorganized, randomly oriented collagen fibers staining blue-green, indicative of immature fibrous tissue rather than organised bone matrix, partially demarcating HB from NB and reflecting ongoing but incomplete bone maturation. Osteocytes were present within NB but the HB-NB boundary remained distinct. Implant A at the same timepoint presented a different pattern: predominantly red staining was observed throughout the peri-implant region, with reduced blue-green FT, and NB displayed well-organized mineralized matrix in closer apposition to the IS (Figure 7A(d–f)). The HB-NB interface was less distinct, Ocy were abundant within NB, BM spaces were clearly developed, and FT was minimal, collectively suggesting that the bioactive coating may have contributed to earlier maturation of newly formed peri-implant tissue from fibrous to mineralized collagen within the same observation window.

By 9 weeks, Implant B showed progressive mineralization with increased red staining compared to the 5-week timepoint, though discrete blue-green FT persisted at the BII between the IS and mineralized bone (Figure 7B(a–c)), consistent with the residual FT identified on H&E. Partial LB organization was beginning to form within NB. Implant A at 9 weeks showed advanced tissue organization: intense and homogeneous red staining dominated the peri-implant region with virtually absent blue-green FT (Figure 7B(d–f)), HB and NB integration appeared seamless, and NB displayed mature LB with well-organized parallel collagen fiber orientation directly adjacent to the IS, consistent with the organized lamellar collagen arrangement confirmed on H&E. Abundant embedded Ocy and well-developed BM spaces were also observed. The Gomori Trichrome findings suggest that Implant A showed favorable collagen remodeling and an earlier transition from fibrous to organized mineralized bone compared to Implant B at both timepoints, though these observations remain preliminary given the exploratory nature of this study.

#### 3.6.3. Calcium Deposition and Mineralization Assessment by Alizarin Red S Staining

**Figure 8:**
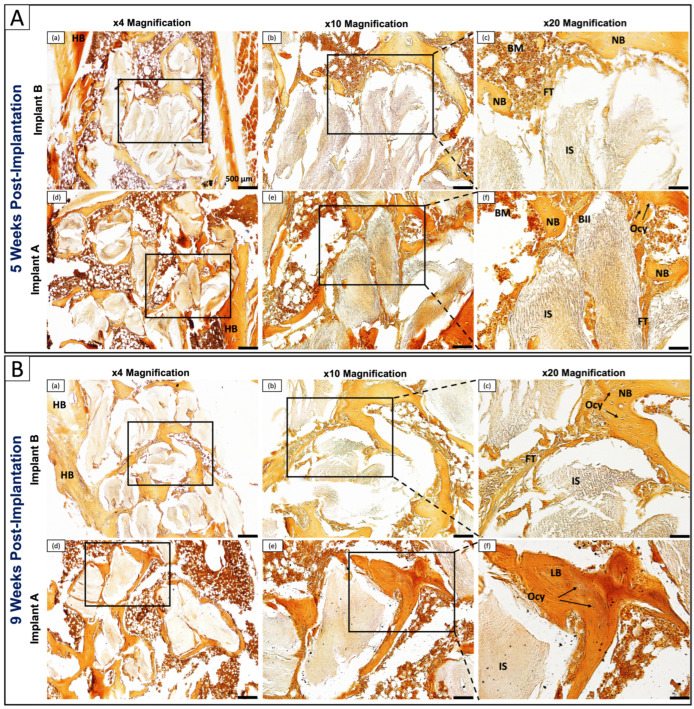
Histological evaluation of bone–implant interface following implantation of Implant A and Implant B 3D-printed interference screw heads in the proximal tibial epiphysis of rats. Representative Alizarin Red S-stained sections are shown at low (×4), intermediate (×10), and high (×20) magnifications. Mineralized regions appear red following Alizarin Red S staining. (**A**) Five weeks post-implantation: panels (**a**–**c**) correspond to Implant B, and panels (**d**–**f**) correspond to Implant A implants. (**B**) Nine weeks post-implantation: panels (**a**–**c**) correspond to Implant B, and panels (**d**–**f**) correspond to Implant A. Black rectangles indicate regions shown at higher magnification. Abbreviations: IS, interference screw; FT, fibrous tissue; NB, newly formed bone; LB, lamellar bone; BII, bone–implant interface; Ocy, osteocytes within lacunae; BM, bone marrow; MnC, mononuclear inflammatory cells; blood sinusoids are indicated by asterisks (*). Observation timepoints: 5 weeks (**A**) and 9 weeks (**B**) post-implantation. Scale bar: 500 μm.

To directly visualize calcium deposition at the bone–implant interface, and provide an independent mineralization readout complementary to the morphological and collagen organization data, Alizarin Red S staining was performed at 5 and 9 weeks (Figure 8). Mineralized regions appear as intense red-orange staining.

At 5 weeks, Implant B showed moderate and heterogeneous mineralization in the peri-implant region, with patchy staining around HB and relatively lighter staining in NB compared to mature HB, suggesting ongoing but incomplete mineralization (Figure 8A(a–c)). FT with minimal mineralization persisted at the interface, while NB displayed characteristic trabecular architecture with BM spaces interspersed between mineralized trabeculae; the trabecular pattern was consistent with woven bone, with irregular mineralised spicules and incompletely formed trabeculae. Ocy were embedded within NB, and HB/NB integration was partial, consistent with early-stage osseointegration. Implant A at the same timepoint showed enhanced mineralization, with intense and homogeneous red-orange staining throughout the peri-implant region (Figure 8A(d–f)). The BII showed direct apposition of mineralized bone to the IS with minimal intervening FT, and NB displayed robust staining with Ocy clearly visible within lacunae, surrounded by densely mineralized matrix and well-integrated BM spaces, suggesting advanced mineralization at this early timepoint.

By 9 weeks, Implant B showed progressive maturation with increased and uniform staining intensity compared to the 5-week timepoint, though staining in NB remained slightly lower than that of mature HB, and FT persisted at the immediate interface (Figure 8B(a–c)), consistent with findings from both H&E and Gomori Trichrome staining. NB had matured substantially with embedded Ocy surrounded by well-mineralized matrix, and trabecular architecture with BM spaces was maintained, reflecting continued but incomplete bone maturation. Implant A showed advanced mineralization across all magnifications, with extensive homogeneous staining approaching the intensity of mature HB throughout the peri-implant region (Figure 8B(d–f)). NB transitioned into LB with well-organized parallel lamellae directly adjacent to the IS, with a dense and homogeneous mineralisation pattern consistent with mature lamellar bone architecture confirmed across all three staining modalities. Abundant embedded Ocy and well-developed BM spaces were present, indicating advanced bone remodeling fully corroborating the lamellar architecture identified on H&E and Gomori Trichrome.

All in all, qualitative assessment of Alizarin Red S staining revealed that both Implant A and Implant B exhibited progressive calcium deposition and mineralization from 5 to 9 weeks. These results suggest that while progressive mineralization occurs in both conditions, Implant A showed a trend toward accelerated and extensive mineralization compared to Implant B, complementing the overall osseointegration findings across all staining modalities.

Considered together, the three stainings produced complementary findings across both timepoints, establishing a coherent and internally consistent picture of osseointegration. H&E revealed the cellular and architectural basis of bone–implant integration; Gomori Trichrome demonstrated the accelerated transition from fibrous to mineralized collagen matrix; and Alizarin Red S directly confirmed enhanced calcium deposition. Each modality reinforced the others, and the temporal progression observed in Implant A from a trend toward enhanced NB formation with reduced FT at 5 weeks to mature LB with direct bone–implant contact at 9 weeks is consistent with the earlier osteogenic marker expression observed in vitro (ALP and BGLAP upregulation at day 14), supporting the biological coherence of the overall dataset while acknowledging the preliminary and exploratory nature of these findings.

## 4. Discussion

This study demonstrates proof-of-concept for a 3D-printed PCL + PLGA + HA composite interference screw with a modular bioactive surface coating, providing complementary in vitro and in vivo evidence of its biocompatibility and preliminary osteogenic potential. The progressive and logically interconnected experimental design from material selection through biological validation to in vivo osseointegration assessment establishes a coherent mechanistic narrative from the material level to the tissue level, each experimental step building directly upon the preceding findings.

### 4.1. Composite Material Selection and Synergistic Biocompatibility

The selection of PCL + PLGA + HA (30/30/40) as the optimal formulation was supported by Alamar Blue and SEM data. At day 21, MSC metabolic activity on PCL + PLGA + HA was more than double than that measured on PCL + HA, and significantly exceeded that of PCL + PLGA, demonstrating the clearest and most consistent support for MSC proliferation across the entire culture period. This superior performance could reflect a synergistic interaction among the three components: PCL contributes long-term mechanical stability and slow degradation [93], PLGA enhances porosity and surface hydrophilicity to facilitate cellular access and metabolite exchange [94], and HA provides osteoconductive nucleation sites that directly promote cell anchorage and osteoblastic priming [95]. The surface coating strategy employed here relies on cell-contact-dependent bioavailability within a polyelectrolyte multilayer, in which the bioactive molecule is presented at the coating surface and made biologically available upon direct cell-surface interaction, as previously described [71,72]. This approach is fundamentally distinct from passive diffusion or burst-release delivery systems and was designed specifically to circumvent the dose-dependent adverse effects associated with uncontrolled high-dose administration.

SEM morphology at high magnification confirmed robust cell spreading, filopodial extension, and three-dimensional scaffold infiltration without morphological abnormalities, with the characteristic elongated cell shape indicative of proliferative and synthetic phases, findings consistent with strong cell–substrate interactions. The Alamar Blue, SEM, and subsequent in vivo histological data allows high-confidence affirmation of the composite’s cytocompatibility, a fundamental prerequisite for clinical translation and a finding that directly justifies progression toward advanced preclinical evaluation. The mechanical properties of PCL/PLGA/HA composite scaffolds at similar compositions are well established in the published literature [48,96,97,98,99,100,101,102]. Compressive moduli and tensile properties compatible with trabecular bone have been reported for FDM-fabricated PCL/HA constructs [101,102], and Wei et al. demonstrated compressive strength exceeding 40 MPa for 3D-printed HA/PLGA scaffolds at 45% HA content [96], confirming the mechanical suitability of HA/PLGA composites at similar ratios. The nanometric surface coating does not alter the mechanical behavior of the underlying matrix. The present study was designed as a proof-of-concept biological investigation and accordingly does not include device-level biomechanical characterization. While no data on pull-out strength, insertion torque, compressive strength, or fatigue resistance are reported here, biomechanical testing of full clinical-size screws under physiologically relevant loading conditions is designated as a primary endpoint of the next translational phase in a porcine model and represents a necessary step before any further progression of this system. The precise matching of PCL, PLGA, and HA proportions to the resorption timeline of the target tissue will however be an important parameter to optimize in future large animal studies. Formal degradation characterization, including mass loss, molecular weight evolution, and pH measurement under physiological conditions, was not performed in the present study and represents a recognized limitation. The ternary composition was nevertheless designed with complementary degradation profiles in mind: PCL provides slow degradation over several months to years supporting sustained mechanical integrity during the critical early fixation period [45], while PLGA degrades more rapidly due to the higher hydrophilicity of glycolic acid residues, creating progressive in situ porosity to facilitate cellular infiltration and matrix remodeling at later implantation stages [46]. The degradation behavior of both polymers and their composites is extensively documented in the published literature [45,46,53,103,104], and reproducing this established dataset was not considered scientifically indicated at the proof-of-concept stage. Degradation kinetics and their correlation with bone formation at matched timepoints are designated as primary endpoints of the planned porcine model study, to be conducted under dynamic loading conditions at clinically relevant timepoints. Complementary physicochemical characterization of the PCL, PLGA, and HA components, including FTIR confirmation of functional groups, XRD phase identification of hydroxyapatite crystallinity, and TGA/DSC thermal analysis, was not repeated in the present study given the well-established physicochemical profiles of these commercial materials at similar compositions in the published literature [46,48,52,96,98,105,106,107,108]. Multiple independent studies employing FTIR and XRD characterization of FDM-fabricated PCL/HA and PCL/PLGA/HA composites have confirmed the absence of new chemical bond formation during processing, and thermal analyses using Resomer-grade PLGA have confirmed stability at FDM processing temperatures [106,107,108]. Water contact angle, surface roughness quantification, and formal BET porosity analysis were not performed in the present study and represent acknowledged limitations of this proof-of-concept work, planned for the material optimization phase of the large-animal study.

### 4.2. Modular Surface Coating Bioavailability and Osteoinductive Signaling

SEM characterization confirmed successful deposition of the surface coating as a discontinuous particulate layer with nodular morphology at ×3500 magnification, consistent with previously reported data by our group and others to enable cell-contact-dependent bioavailability while preserving surface porosity [71,73,79,90]. In this system, the bioactive molecule is not freely released into the surrounding medium but is made available through direct cell-surface contact, which represents a key safety advantage over conventional high-dose delivery strategies. This structural characterization was an essential prerequisite for meaningful interpretation of the RT-qPCR and in vivo results, as it established that observed biological effects could be attributed to the coating rather than non-specific surface modifications. This approach is consistent with the broader evidence base for polyelectrolyte multilayer coatings in orthopedic osseointegration applications [62].

The RT-qPCR data provided functional evidence that the bioactive surface coating retained biological activity. The significant upregulation of ALP and BGLAP in Implant A at day 14 (*p* < 0.001 for both) is consistent with the well-characterized early-phase osteogenic cascade: ALP peaks during matrix maturation and precedes mineralization [90,91], while BGLAP encodes osteocalcin, directly involved in hydroxyapatite binding and a marker of committed osteoblastic activity. Notably, this selective early upregulation reflects the physiologically constrained nature of the cell-contact-dependent coating strategy, consistent with the localized biological availability intended by this approach [40,41]. RUNX2 expression was elevated in Implant B controls at day 14 with no significant difference between groups at day 21. One possible explanation is a temporal offset: RUNX2 is transiently induced early in the osteogenic cascade [90], and in Implant A its peak may have preceded the day 14 sampling point, consistent with the earlier ALP and BGLAP upregulation observed in the same group. By day 21, all markers reached comparable levels across both conditions, coherent with the natural kinetics of osteogenic differentiation [91,92] and consistent with the progressive mineralization and lamellar bone formation subsequently observed histologically at both in vivo timepoints. This molecular-to-tissue continuity, from transcriptional osteoblastic activation at day 14 in vitro to enhanced calcium deposition at 5 weeks in vivo, establishes the biological coherence of the overall dataset and supports a mechanistic link between the coating’s osteoinductive activity and the enhanced osseointegration observed in vivo. While direct protein-level confirmation of the in vitro RT-qPCR findings was not performed in the present study, the in vivo Alizarin Red S and H&E data provide complementary tissue-level evidence consistent with functional osteogenic activity; Western blotting and immunofluorescence staining for osteogenic markers are planned in the dedicated material optimization phase of the large-animal study.

### 4.3. In Vivo Biocompatibility and Progressive Osseointegration

The in vivo results provide direct relevant evidence supporting the potential of this system. The primary objective of this in vivo experiment was to establish the biocompatibility and safety profile of the composite implant in a living system, in compliance with the 3R principles, using the minimum number of animals sufficient to obtain preliminary histological evidence. The absence of cyst formation, osteolysis, and pathological fibrosis across all animals, both conditions, and both timepoints represents an important preliminary safety finding, suggesting that neither the composite material nor the surface coating strategy induced adverse local tissue reactions at the dose evaluated. These results are encouraging but should be confirmed in larger, adequately powered preclinical studies. The limited inflammatory response, restricted to physiological MnC, is consistent with the well-established biocompatibility of PCL and PLGA in orthopedic applications [53,109,110] and directly corroborates the in vitro Alamar Blue and SEM data, completing a consistent safety narrative that spans from cell culture to the living organism.

The three complementary histological staining modalities produced mutually reinforcing findings that together paint a coherent picture of osseointegration progression. H&E revealed the cellular and architectural basis of bone–implant integration; Gomori Trichrome demonstrated the accelerated transition from immature fibrous to organized mineralized collagen matrix; and Alizarin Red S directly confirmed enhanced calcium deposition with each modality independently supporting and strengthening the conclusions drawn from the others.

At 5 weeks, Implant B displayed the characteristics of early phase osseointegration: woven bone formation, FT interposition at the BII, and moderate, heterogeneous mineral deposition. Implant A at the same timepoint demonstrated advanced integration: reduced FT, closer NB apposition to the IS, organized BM spaces, and homogeneous and intense calcium deposition, suggesting a potential contribution of the bioactive coating to the early bone formation response. By 9 weeks, this divergence was further amplified: Implant A had transitioned to mature LB with well-organized parallel collagen lamellae in direct contact with the IS, virtually absent FT, and mineral density approaching that of native host bone, while Implant B showed continued but incomplete maturation with persistent FT. The temporal progression from woven bone at 5 weeks to lamellar bone at 9 weeks in both groups confirms that the composite material itself supports physiological osseointegration, with Implant A showing advanced progression through this process. This temporal divergence is consistent with the bioactive coating contributing to an accelerated woven-to-lamellar bone transition and supports a biological link between the earlier osteogenic marker expression observed in vitro and the advanced tissue-level integration observed in vivo. Given the exploratory design and limited sample size (n = 2 per group per timepoint), these histological observations are reported as qualitative and preliminary findings only. The rat tibial epiphysis model and implantation of the screw head only were selected to provide initial biological evidence in a well-controlled setting, and all findings should be interpreted within this context rather than as a direct model of ACL reconstruction.

### 4.4. Translational Perspectives and Future Directions

To contextualize the present system within the existing landscape of interference screw and bioactive fixation technologies, Table 1 below summarizes representative commercial and research-stage devices according to material composition, fabrication method, and bioactive coating strategy. Current commercial interference screws, whether PEEK-based or bioabsorbable PLLA/PGA, provide immediate mechanical fixation but carry no bioactive surface functionality [111]. Osteoconductive coatings such as HA or calcium phosphate surface treatments represent a first step toward biological augmentation but do not actively stimulate osteogenic differentiation [112,113]. Biocomposite screws incorporating β-TCP, such as the Milagro device, improve osteoconductive properties but rely on passive resorption rather than active cellular signaling [114,115]. More recent research-stage approaches have explored 3D-printed titanium screws with calcium phosphate surface modification and magnesium-based screws that indirectly promote BMP-2 accumulation, demonstrating the field’s progression toward biologically active fixation systems [113,116,117]. BMP-2 surface immobilization strategies have been reported for artificial ligament surfaces using polydopamine-mediated coating, though this approach relies on passive release rather than cell-contact-dependent bioavailability [118]. The only existing 3D-printed polymer composite interference screw incorporating HA/PLA/PCL was fabricated without any bioactive coating and characterized mechanically only, without biological validation [119]. The present system is therefore distinguished by the combination of FDM fabrication of a ternary PCL + PLGA + HA composite screw geometry with a cell-contact-dependent BMP-2 nanoreservoir coating, supported here by a multi-level proof-of-concept biological evaluation. These observations should be interpreted within the limitations of the proof-of-concept design, and the translational relevance of this comparison will be fully established in the planned large-animal phase.

This study was designed as a proof-of-concept investigation, with biocompatibility assessment and preliminary osseointegration evaluation as primary objectives, conducted in strict compliance with the 3R principles using the minimum number of animals required. The rat tibial epiphysis model provided an appropriate and ethically justified site for this initial evaluation, though miniaturization requirements limited implantation to the screw head only. The next translational step will involve biofabrication of full clinical-size screws implanted according to current surgical standards in a porcine model, which closely approximates human bone anatomy and implant size requirements for clinical-grade interference screws, allowing biomechanical pull-out and fixation strength testing under physiologically relevant loading conditions, with adequately powered sample sizes to enable statistically robust analysis.

Quantitative bone histomorphometry, including bone volume fraction, bone–implant new bone area, fibrous tissue thickness, and trabecular thickness, was not performed in this proof-of-concept study given the exploratory design and limited sample size, and is designated as a primary endpoint of the planned porcine study, where full clinical-size screw geometry, adequate sample sizes, and standardized measurement protocols will allow meaningful and statistically robust assessment of osseointegration quality.

An important translational consideration is the cost-effectiveness of the 3D-printed composite screw relative to existing commercial alternatives. As summarized in Table 1, current commercial interference screws, including PEEK-based devices and bioabsorbable PLGA/β-TCP screws, are manufactured by injection molding or precision machining, processes involving dedicated tooling and established regulatory pathways. Health technology assessment modeling has confirmed that improved biological and mechanical performance of bioabsorbable implants in ACL reconstruction generates positive cost–benefit outcomes [121]. The FDM 3D printing approach described here uses commodity PCL, PLGA, and hydroxyapatite and does not require dedicated tooling, offering manufacturing flexibility and cost advantages over conventional methods, with FDM recognized as more cost-effective than alternative 3D printing technologies and offering patient-specific customization without additional tooling costs [61]. The modular BMP-2 nanoreservoir coating uses a simple dip-coating process scalable to batch production. A formal cost-effectiveness analysis incorporating manufacturing scale-up, sterilization, regulatory compliance, and comparative clinical outcome modeling is beyond the scope of this proof-of-concept study and will be incorporated into the health economic evaluation planned to accompany the large-animal preclinical study.

The absence of adverse tissue reactions at the dose used is encouraging in the context of known safety concerns associated with high-dose administration [40,41] and provides a rational basis for considering cell-contact-dependent surface coating as a safer and more localized alternative. Beyond the molecule used here, the coating bioavailability is inherently modular and adaptable, anti-inflammatory agents, antimicrobial peptides, and angiogenic factors such as vascular endothelial growth factor (VEGF) could be incorporated, to simultaneously address multiple biological challenges of graft–bone healing. This modularity positions the composite screw platform as a versatile foundation for next-generation regenerative fixation devices.

## 5. Conclusions

This study establishes proof-of-concept for a 3D-printed PCL + PLGA + HA composite interference screw with a modular bioactive surface coating as a biocompatible and osteoconductive fixation platform. Starting from a systematic comparison of three polymer formulations, the PCL + PLGA + HA (30/30/40) formulation demonstrated superior support for MSC metabolic activity in vitro, confirmed by SEM morphology showing robust cell attachment, spreading, and three-dimensional scaffold infiltration without cytotoxic effects. The modular bioactive surface coating was successfully deposited on Implant A and was associated with earlier upregulation of key osteogenic markers (ALP and BGLAP), consistent with an acceleration of the early osteoblastic transcriptional program and the established temporal dynamics of osteogenic differentiation.

These in vitro findings were directly reflected in vivo. All implants demonstrated biocompatibility across 5 and 9 weeks, with no adverse tissue reactions in either coating condition. Qualitative histological assessment across three complementary staining modalities indicated a trend toward earlier and advanced osseointegration in Implant A, with reduced fibrous tissue interposition, denser calcium deposition, and mature lamellar bone architecture at the bone–implant interface compared to Implant B. While the biological proof-of-concept data presented here are encouraging, the absence of device-level biomechanical characterization, including pull-out strength, insertion torque, and fatigue resistance, represents a recognized limitation of the present work. Such validation is designated as a primary objective of the planned large-animal study.

Taken together, these preliminary observations are encouraging and provide a solid biological foundation for the next phase of this research, which will involve evaluation of full clinical-size screws in a porcine model, with adequate sample sizes to allow statistically powered analysis, and with dedicated funding already secured to support this translational continuation. The inherently modular nature of this composite biomaterial further opens the door to multifunctional coatings combining osteogenic, angiogenic, and immunomodulatory signals, positioning this system as a versatile foundation for next-generation regenerative fixation devices with the potential to contribute to improved biological integration and fixation outcomes in orthopedic reconstruction.

## Figures and Tables

**Table 1:** Comparison of representative existing commercial and research-stage interference screw and fixation systems with the present study. FDM: fused deposition modeling; HA: hydroxyapatite; PCL: polycaprolactone; PLGA: poly(lactic-co-glycolic acid); PEEK: polyetheretherketone; CaP: calcium phosphate; TCP: tricalcium phosphate; HP Mg: high-purity magnesium.

Device/System	Material	Fabrication	Coating	References
PEEK screws (Arthrex, Smith & Nephew)	PEEK polymer	Injection molding	None	[111]
PLLA/PGA screws (commercial bioabsorbable)	Absorbable polymer blend	Injection molding	None	[120]
PLGA/β-TCP screw (Milagro, DePuy Mitek)	PLGA 70% + β-TCP 30%	Injection molding	Osteoconductive TCP only	[114,115]
PLA/HA bioabsorbable screw	Modified mPLA + HA	Injection molding	Osteoconductive HA only	[112]
3D-printed Ti alloy screw + CaP surface	Ti-6Al-4V + CaP coating	Additive manufacturing + chemical treatment	Osteoconductive CaP only	[113,117]
HP Mg interference screw	High-purity magnesium	Machining	Indirect BMP-2/VEGF accumulation	[116]
BMP-2 on PET artificial ligament	PET + polydopamine	Weaving/Braiding	BMP-2 via polydopamine coating (sustained surface release)	[118]
PCL/HA scaffold	PCL + HA	3D solid freeform fabrication	None	[55]
PLGA/HA scaffold	PLGA + HA	Gas foaming/ particulate leaching	None	[51]
HA/PLA/PCL 3D-printed screw	HA + PLA + PCL	FDM 3D printing	None	[119]
BMP-2 collagen sponge (InFUSE^®^)	Absorbable collagen	Lyophilization	BMP-2 (burst release, high dose)	[70]
Present study (Implant A)	PCL + PLGA + HA (30/30/40)	FDM 3D printing	BMP-2 nanoreservoir (cell-contact-dependent)	Present study

## Data Availability

The data supporting the findings of this study are available within the article. Additional data are available from the corresponding authors upon reasonable request.

## Abbreviations

The following abbreviations are used in this manuscript:

ACL	Anterior cruciate ligament
ALP	Alkaline phosphatase
ANOVA	Analysis of variance
BII	Bone–implant interface
BGLAP	Bone gamma-carboxyglutamate protein
BM	Bone marrow
BMP-2	Bone morphogenetic protein-2
BSPII	Bone sialoprotein II
BV/TV	Bone volume fraction
CO_2_	Carbon dioxide
EDTA	Ethylenediaminetetraacetic acid
FDA	U.S. Food and Drug Administration
FDM	Fused deposition modeling
FT	Fibrous tissue
GAPDH	Glyceraldehyde 3-phosphate dehydrogenase
H&E	Hematoxylin and eosin
HA	Hydroxyapatite
HB	Host bone
hBM-MSC	Human bone marrow-derived mesenchymal stem cell
HMDS	Hexamethyldisilazane
IS	Interference screw
KOOS	Knee Injury and Osteoarthritis Outcome Score
LB	Lamellar bone
MES	2-(N-morpholino)ethanesulfonic acid
MnC	Mononuclear inflammatory cells
MSC	Mesenchymal stem cell
NB	Newly formed bone
OCN	Osteocalcin
Ocy	Osteocyte
PBS	Phosphate-buffered saline
PCL	Polycaprolactone
PLGA	Poly(lactic-co-glycolic acid)
PRP	Platelet-rich plasma
RT-qPCR	Reverse transcription quantitative polymerase chain reaction
RUNX2	Runt-related transcription factor 2
SD	Standard deviation
SE	Standard error of the mean
SEM	Scanning electron microscopy
α-MEM	Alpha-minimum essential medium
VEGF	Vascular endothelial growth factor
